# Emerging resistance *vs.* losing response to immune check point inhibitors in renal cell carcinoma: two differing phenomena

**DOI:** 10.20517/cdr.2023.47

**Published:** 2023-09-20

**Authors:** Arya Mariam Roy, Saby George

**Affiliations:** Division of Hematology and Oncology, Department of Internal Medicine, Roswell Park Comprehensive Cancer Center, Buffalo, NY 14203, USA.

**Keywords:** Renal cell carcinoma, immunotherapy, immune checkpoint inhibitors, primary resistance, acquired resistance, immune exhaustion markers, immunosuppressive tumor microenvironment

## Abstract

The introduction of immune checkpoint inhibitor (ICI) has revolutionized the treatment of metastatic renal cell carcinoma (mRCC) and has dramatically improved the outcomes of patients. The use of monotherapy or combinations of ICIs targeting PD-1/PD-L1 and CTLA-4, as well as the addition of ICIs with tyrosine kinase inhibitors, has significantly enhanced the overall survival of mRCC patients. Despite these promising results, there remains a subset of patients who either do not respond to treatment (primary resistance) or develop resistance to therapy over time (acquired resistance). Understanding the mechanisms underlying the development of resistance to ICI treatment is crucial in the management of mRCC, as they can be used to identify new targets for innovative therapeutic strategies. Currently, there is an unmet need to develop new predictive and prognostic biomarkers that can aid in the development of personalized treatment options for mRCC patients. In this review, we summarize several mechanisms of ICI resistance in RCC, including alterations in tumor microenvironment, upregulation of alternative immune checkpoint pathways, and genetic and epigenetic changes. Additionally, we highlight potential strategies that can be used to overcome resistance, such as combination therapy, targeted therapy, and immune modulation.

## INTRODUCTION

Renal cell carcinoma (RCC) accounts for approximately 3% of all adult malignancies^[1]^. The incidence of RCC has been steadily increasing with 81,800 new cases estimated to be diagnosed in the United States in 2023^[2]^. The 5-year relative survival rate has increased over time, with 72% survival for patients with locoregional disease, but the survival for metastatic RCC is still poor: 15%^[2]^. There are different types of RCC with diverse clinical and epigenetic characteristics, of which clear cell type is the most common variant (80%), followed by papillary (15%) and chromophobe (3%-5%) histological variants^[3]^.

The main therapeutic options in metastatic RCC (mRCC) were interferons and interleukins till 2006. Significant advancements have been made in the field of RCC with the development of vascular endothelial growth factor receptors (VEGFR) inhibitors and mammalian target of rapamycin (mTOR) inhibitors^[4-6]^. RCC has been recognized as an immunosuppressive disease. With the advent of novel immune checkpoint inhibitor (ICI) targeting programmed death-1 (PD-1), programmed death ligand-1 (PD-L1) and cytotoxic T-lymphocyte associated protein-4 (CTLA-4), the treatment paradigm of RCC has changed tremendously^[4,7,8]^.

Currently, ICI monotherapy and ICI combination with TKI/ICI are the standard of treatment in advanced RCC, which have significantly improved the survival of treatment-refractory mRCC patients. Nivolumab, an anti-PD-1 antibody, was approved for use in mRCC in 2015 based on the overall survival benefit seen in the CheckMate 025 trial. The objective response rate (ORR) with nivolumab was 25% compared to 5% with everolimus^[9]^. Following this, based on the results of the CheckMate 214 trial, the combination of nivolumab and ipilimumab was approved for use in treatment-naive intermediate and poor-risk mRCC patients [International Metastatic Renal Cell Carcinoma (IMDC) risk stratification criteria]^[10]^. The combination has resulted in an improvement in overall survival, with an ORR of 42% and a complete response rate of 11% over a span of 5 years^[11]^. Several VEGFR-TKIs have shown survival advantages in mRCC when used in combination with the ICI. The combination of pembrolizumab (PD-1 inhibitor) and axitinib (VEGFR inhibitor) (Keynote-426), nivolumab and cabozantinib (CheckMate 9ER), lenvatinib and pembrolizumab (CLEAR trial), avelumab (PD-L1 inhibitor) and axitinib (JAVELIN Renal 101) are approved as first-line options for patients with mRCC^[12-15]^. All these treatments have resulted in a superior response rate and progression-free survival (PFS) compared to comparator, sunitinib.

Although these treatments have shown long-term clinical benefits in a large fraction of patients, some patients have progressive disease as the best response, consistent with primary resistance. Some patients progress after responding for a certain period, reflecting acquired resistance to these systemic treatment options. All these treatments have improved the outcomes in mRCC; however, these are limited by the innate and acquired resistance that emerges. The cancer-immunity cycle, which involves the development of neoantigens, antigen presentation, T-cell responses, and the recognition and destruction of cancer cells, describes the inherent immune biology of cancer and resistance to ICIs^[16]^. Understanding the mechanisms behind the resistance is needed to develop therapeutic strategies to overcome it and thus maximize therapeutic efficacy. In this review article, we mention the mechanism of action of ICI, highlight the mechanisms of resistance to ICI, and the potential approaches to overcome resistance to ICI in mRCC.

### Mechanism of action of immune checkpoint inhibitors

**Figure 1:**
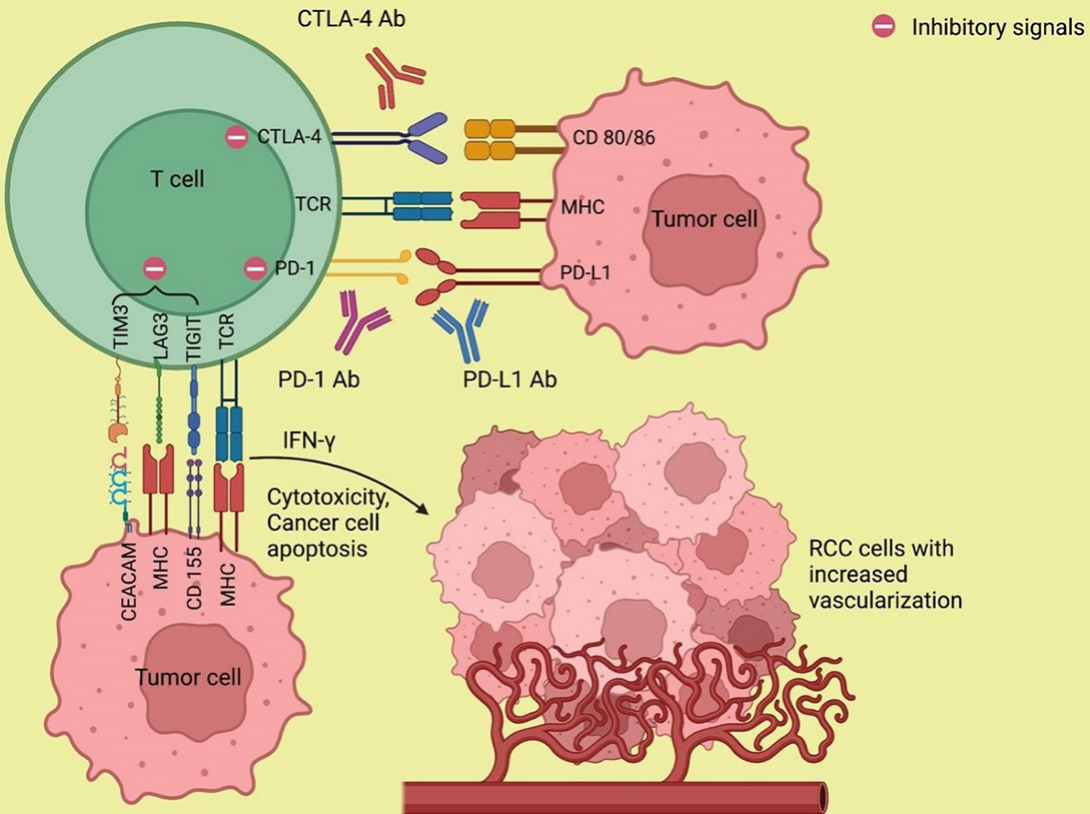
Mechanism of action of immune checkpoint inhibitors. The monoclonal antibodies against PD-1/PD-L1 and CTLA-4 abolish the T-cell inhibitory responses that are stimulated by the interaction of PD-1 with PD-L1 and CTLA-4 with its ligand CD 80/86 and thereby enhance antitumor immunity. Ab: Antibody; CD 80/86: cluster of differentiation 80/86; CEACAM: carcinoembryonic antigen-related cell adhesion molecule; CTLA-4: cytotoxic T lymphocyte-associated protein 4; IFN-γ: interferon-γ; LAG3: lymphocyte-activation gene 3; MHC: major histocompatibility complex; PD-1: programmed death-1; PD-L1: programmed death ligand-1; RCC: renal cell carcinoma; TCR: T cell receptor; TIGIT: T cell immunoglobulin and ITIM domain; TIM3: T cell immunoglobulin and mucin domain 3. (Figure credits: Roy AM).

The mechanism of resistance to ICI is not completely understood. To understand the biology behind the resistance formation, an adequate understanding of the mechanism of action of immunotherapy is necessary. The major histocompatibility complex (MHC)/antigen on the antigen-presenting cells (APCs) interacts with the T cell receptors (TCR) and activates the T cells which lead to a cascade of events involving stimulatory and inhibitory signals^[17]^. Upon activation, the T cells release interferon-gamma (IFN-γ), which promotes cytotoxicity and results in the upregulation of PD-L1 expression in the tumor cells. The PD-1, which is expressed on the activated T cells, interacts with the PD-L1, resulting in the inhibition of the antitumor response by the T cells^[18]^. Similarly, the interaction of CTLA-4 expressed on the T cells with its ligands CD80/CD86, B7 on APC prevents the stimulation, proliferation, and activation of T cells, thus diminishing the immune response^[19,20]^. The above is responsible for T cells being anergic and relatively inactive against certain cancers like RCC and melanoma. Expression of other coinhibitory receptors, such as TIM-3 and LAG-3, leads to T-cell exhaustion^[21]^. ICIs targeting PD-1/PD-L1, and CTLA-4 can facilitate T-cell activation and overcome anergy and thus improve the antitumor immune response^[7]^ [Figure 1].

### Mechanism of resistance to immunotherapy in RCC

**Figure 2:**
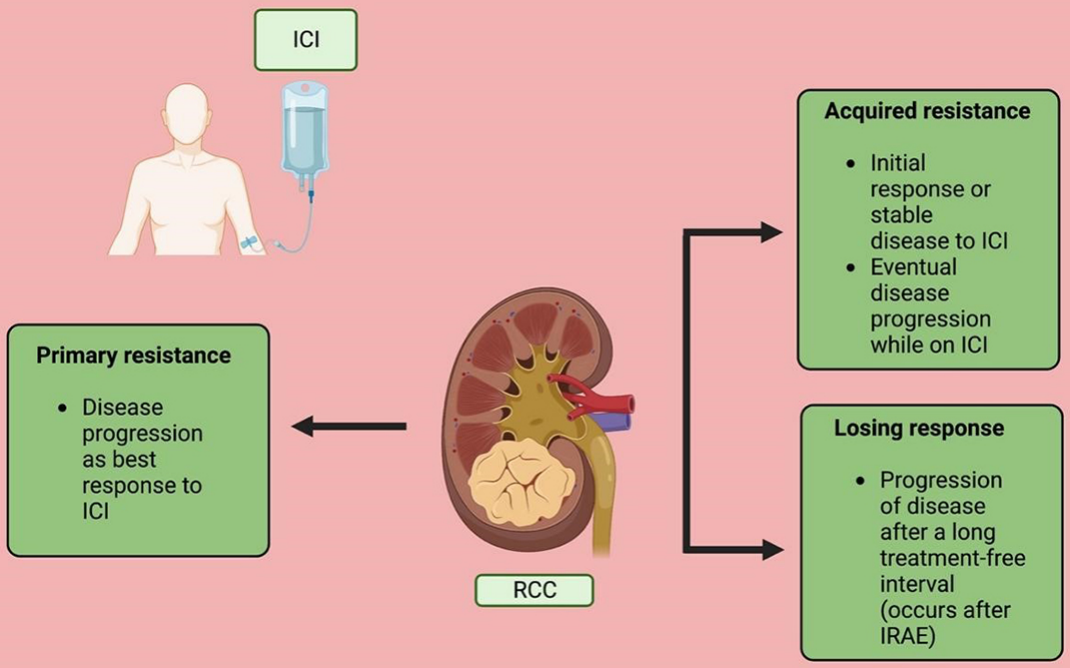
Types of resistance. ICI: Immune checkpoint inhibitor; IRAE: immune-related adverse events; RCC: renal cell carcinoma. (Figure credits: Roy AM).

The resistance to ICI occurs due to the complex and evolving interactions between the immune system and cancer cells. Several patient factors, tumor microenvironment factors, and oncogenic signaling pathways play an essential role in the development of resistance to systemic therapies. The resistance to immunotherapy can be classified broadly into two categories: (a) primary resistance, in which patients will have progressive disease as best response to immunotherapy; and (b) acquired resistance, in which patients will respond to immunotherapy for some time and eventually have progression of the disease. This could occur in two ways: (a) resistance formation while on the drug (acquired resistance) and (b) progression of disease after a long treatment-free interval, which is uniquely seen after discontinuation of therapy upon the development of immune-related adverse events (IRAE) (we call it “loss of response”) [Figure 2]. There is no specific consensus regarding the timeline of the development of the acquired resistance^[21]^. High tumor mutational burden (TMB) and high neoantigen expression have been associated with a more consistent response to ICI^[22]^. Resistance to ICI therapy can be due to a defect in any of the steps explained in the mechanism of action of ICI. It could be due to insufficient production or impaired function of antitumor T-cells, or lack of adequate memory T-cell formation. Several patient factors, tumor microenvironment (TME) factors, oncogenic pathways, and immune checkpoints impact the creation and sustainment of an antitumor microenvironment.

### Tumor microenvironment

TME in RCC is composed of several factors, such as extracellular matrix, immune cells, stromal cells, aberrant blood vessels, cytokines, and growth factors, which affect the growth, development and progression of the tumor and the treatment response. RCC is one of the tumors which has a high immune microenvironment composed of T cells. Increased proportion of the T regulatory cells (Tregs) in the TME is associated with an immunosuppressive environment in several malignancies^[23]^. Tregs suppress the T effector (T-eff) cells through inhibitory cytokines such as transforming growth factor (TGF)-β, interleukin (IL)-10, direct cytotoxicity through perforins/granzyme, promotion of T cell exhaustion and thus prevent the tumor-specific immune response which leads to resistance to ICI^[24-26]^. The proportion of Tregs has predictive and prognostic values^[27-29]^. In a study by Griffiths *et al.*, a high frequency of Tregs in the peripheral blood of RCC patients was found to be associated with reduced survival^[30]^.

Myeloid-derived suppressor cells (MDSCs) in the TME are potent suppressors of various T cell functions, which facilitate tumors to evade immune responses. They regulate T cell proliferation, induce T cell apoptosis, and are involved in the inhibition of MHC class II proteins through several mechanisms such as arginase and nitric oxide production^[31]^. Tumor-associated factors such as TGF-β, platelet-derived growth factors (PDGFs), and Interleukins (IL-3, IL-6, IL-10) induce the production of reactive oxygen species (ROS) by the MDSCs. ROS has been postulated to be one of the mechanisms through which MDSCs inhibit the cytotoxicity of T cells and create an immunosuppressive environment^[32]^. In one of the studies, the depletion of MDSCs reinstituted IFN-γ production and T-cell proliferation in RCC^[33]^. Thus, MDSCs, along with Tregs, create an immunosuppressive tumor environment that resists the activity of ICI.

Another component of the TME that impacts the response to ICI is tumor-associated macrophages (TAMs). During tumor development and progression, macrophages are recruited into the TME and differentiate into mature forms which aid in tumor progression. Macrophages are classified into M1 and M2 subtypes^[34]^. M1 macrophages are associated with inflammatory responses by secreting pro-inflammatory cytokines such as IL-6, IL-12, IL-23, and tumor necrosis factor-α (TNF-α). Nevertheless, the M2 macrophages, especially M2d, employ anti-inflammatory and pro-tumorigenic activities^[35,36]^. Studies have shown that TAMs promote tumor growth, invasion, and metastasis by altering the TME, enhancing angiogenesis and resulting in immune evasion and thus therapeutic resistance^[37-39]^. TAM also has prognostic implications; malignancies with high TAM density have been shown to have poor disease-free survival (DFS) and overall survival (OS)^[40,41]^.

Several cytokines in the TME also play a major role in immunoregulation. The cytokines CCL5, CCL17, CCL22, CXCL8, and CXCL12 promote immunosuppression by recruiting Tregs and MDSCs to the TME^[42]^. Similarly, cytokines also promote antitumor effects, as exhibited by the increased recruitment of cytotoxic T cells (CTLs) to the TME by the cytokines CXCL9 and CXCL10^[43]^. Several growth factors, such as TGF-β, Vascular endothelial growth factor (VEGF), emulate immunosuppression by upregulating Tregs and inhibiting the CTLs which impacts the response to ICI^[44,45]^. By priming the vascular endothelium to allow for the extravasation of Tregs and resistance to the migration of CTLs to the TME, VEGF contributes to immune evasion and promotes rapid tumor growth and progression, while also resulting in resistance to immune checkpoint inhibitors (ICIs).

Single-cell transcriptomic studies and paired single-cell T-cell receptor sequencing have enabled us to comprehend the immune cell composition and the role of T-cell clonotype expansion in response to ICIs^[46,47]^. The study by Braun *et al.* shows progressive immune dysfunction in metastatic RCC, as evidenced by a higher proportion of exhausted T-cells and immunosuppressive M2 macrophages. Additionally, it was observed that TAMs exhibit reduced production of inflammatory cytokines in advanced stages. While their study did not find a higher T-cell exhaustion/TAM interaction signature to be predictive of response to ICI, it was associated with poor overall survival^[46]^. The role of CD8 T-cells in the clinical outcomes of mRCC patients treated with ICI remains controversial. Several studies have linked the infiltration of CD8 T-cells in the tumor microenvironment of RCC patients to a poorer prognosis^[48,49]^. Exploratory data from the JAVELIN RENAL 101 trial revealed an association between high CD8 T cell infiltration and poor PFS in mRCC patients treated with sunitinib^[50]^. This trend was not evident among patients treated with the combination of avelumab + axitinib. In another study by Voss *et al.*, no association was found between CD8 T cell density and clinical response to ICI^[51]^. Hence, based on the current literature, we are unable to definitively establish a correlation between CD8 T cells and treatment response or resistance to ICI therapy, although such a possibility warrants consideration.

### Oncogenic pathways and antigen presentation

Impaired antigen presentation could be associated with ICI resistance. The loss of MHC class molecules results in immune evasion and is known as a mechanism of acquired resistance to ICI. Loss of function of B2-microglobulin results in defective transport of the MHC-1 class molecules and selective downregulation of MHC-1 is associated with resistance to ICI^[52]^. This was evident in a study led by Zaretsky *et al.*, which showed that a truncated mutation in the gene encoding the antigen-presenting protein B2-microglobulin led to the loss of surface expression of MHC-1 molecules and resulted in acquired resistance to PD-1 blockade immunotherapy in melanoma^[53]^.

Several signaling pathways mediate immune responses and contribute to the resistance to immunotherapy. JAK/STAT receptor upregulates MHC-1 expression through IFN-γ signaling which enhances antigen presentation. It also recruits immune cells to the TME and has antiproliferative effects on tumor cells and also enhances apoptosis^[54]^. It was noted that the loss of function mutations in the genes encoding JAK1 or JAK2 resulted in a lack of response to IFN-γ, affects the antiproliferative effects on tumor cells, and thereby affects the response to ICI therapy in malignancies^[53]^. Simultaneously, JAK/STAT receptors also increase PD-L1 expression on tumor cells through interferon regulatory factor 1 (IRF1) which grants resistance to tumor cells to the innate immune system of the body^[55]^. In the setting of ICI targeting PD-1, PD-L1 amplification has shown an improved response to treatment in some malignancies^[56]^. Acquired JAK/STAT mutation results in loss of IFN-γ signaling and leads to resistance to ICI treatments through the inability to upregulate MHC-1 and PD-L1 expression^[53,57]^.

The mitogen-activated protein kinase (MAPK) pathway has been shown to have a significant role in immune evasion. Increased MAPK signaling impairs the recruitment and function of T cells, resulting in immune evasion. MAPK signaling is also involved in the proliferation, apoptosis, and motility of tumors^[58]^. It has been shown that MAPK inhibitors promote cytotoxicity by enhancing IFN-γ signaling, MHC-1 expression, tumor-infiltrating lymphocytes and upregulating PD-L1 expression^[59]^.

The Wnt/β-catenin pathway is overexpressed in many cancers. It takes part in tumorigenesis through the maintenance of cancer stem cells and metastasis and also affects cellular immune regulation^[60]^. Spranger *et al.* demonstrated that the activation and overexpression of the Wnt/β-catenin pathway are associated with resistance to anti-PD-L1 and anti-CTLA-4 monoclonal antibody therapies through T cell exclusion from the microenvironment. They also showed that activation of the WNT/β-catenin signaling pathway results in the absence of a T-cell gene expression signature^[61]^. Loss of phosphatase and tensin homolog (PTEN) leads to the expression of VEGF and activation of the phosphatidylinositol 3-kinase (PI3K) pathway, which aids in tumorigenesis. PTEN deletion also leads to the recruitment of immunosuppressive cytokines in the TME, decreased infiltration of CTLs into tumor sites and has shown inferior outcomes with PD-1 therapy^[62]^.

### Immune checkpoints

PD-1 has been well-known as a marker of T-cell exhaustion. The other relevant immune checkpoints include CTLA-4, T-cell immunoglobulin mucin-3 (TIM-3), and lymphocyte-activation gene 3 (LAG-3), B and T lymphocyte attenuator (BTLA), T-cell immunoreceptor with immunoglobulin and ITIM domains (TIGIT). As mentioned earlier, the exhausted T-cells in advanced RCC exhibit several exhaustion markers, such as PD-1, TIM-3, TIGIT, and CTLA-4^[46,47]^. Cai *et al.* demonstrated that in RCC, TIM-3 expression was associated with infiltration of dysfunctional CTCLs and blockage of TIM-3 pathway in enhanced IFN-γ production and enhanced antitumor immunity^[63]^. It has been demonstrated that the co-expression of these inhibitory immune checkpoints such as PD-1, TIM-3, CTLA-4, and LAG-3 is associated with the progression of lung cancer^[64]^. Expression and upregulation of multiple immune checkpoints lead to T-cell exhaustion and have been linked with acquired anti-PD-1 and anti-CTLA-4 resistance in mouse models and clinically in several malignancies^[64-66]^. Persistence of antigen or chronic exposure to antigen results in over-expression of these inhibitory immune checkpoints and leads to impaired effector T-cell function. Studies have shown that high expression of PD-1 leads to excessive T cell exhaustion and results in poor response to ICI, although tumors with low/intermediate PD-1 expressing CD8 T cells can be stimulated with ICI and lead to therapeutic response. Therefore, a threshold level of PD-1 expression on the intratumor CD8 T-cells can predict treatment response in at least some cancers^[67]^. In addition to its role in the T-cell compartment, CTLA-4 is also reported to play a role in T-cell priming, peripheral tolerance, thymic development, and various other immunological functions that hold potential for therapeutic applications^[68]^.

### Impaired T-cell memory

At least ICI-ICI combination has long-term durable responses as evidenced by the extended follow-up of Checkmate -214, where the combination immunotherapy ipilimumab + nivolumab remained efficacious even after 4 years^[11]^. The effector CD8 T-cells play a pivotal role in the response to ICI as explained. A minority of the effector T cells transform into memory T cells inducing memory and remain inactive until repeated exposure to the antigens, thought to be the reason behind durable responses to dual CTLA-4/PD-1 blockade. It is evident that if the formation of the memory T cells is impaired, it can lead to the loss of response to ICI and will also lead to the development of acquired resistance^[69,70]^. In a study by Ribas *et al.*, it was demonstrated that patients who had poor response to anti-PD-1 therapy had significantly lower tumor-associated memory T cells compared to those who responded^[69]^.

### Other mechanisms of resistance

Given that RCC is a hypervascular tumor, the excessive demand and inadequate supply of nutrients lead to hypoxia, which plays a role in generating an immunosuppressive microenvironment for the tumor to facilitate its progression. This acts as a vicious cycle as hypoxia upregulates genes involved in angiogenesis, cellular proliferation and it aids in the recruitment of immunosuppressive T-regs, MDSCs and accelerates the polarization of macrophages to TAMs, leading to the inhibition of the production and function of CD8 T-cells^[71]^. In addition, the release of hypoxia-induced-factors (HIF) 1 and 2 leads to overexpression of inhibitory checkpoints CTLA4, TIM3, and LAG3 through the generation of VEGF, leading to a more complex tumor microenvironment and thus resistance to ICI^[72]^.

The association of gut microbiome in the response to ICI has been revealed. Analysis of the fecal microbiota sample showed a significant relative abundance of specific species of microbes (Ruminococcaceae family) in the anti-PD-1 responders in melanoma. Enhanced systemic and antitumor immunity was observed in anti-PD-1 responders with favorable gut microbiota. In addition, this response was observed in germ-free mice with fecal transplants from responders. The tumors of mice that received the fecal microbiota transplant from the anti-PD-1 responders had higher CD8 T-cell density compared to the mice that received the transplant from the non-responders^[73]^. The utilization of antibiotics altering the gut microbiota has been shown to have a negative impact on the overall survival of several malignancies including RCC^[74-76]^. Lalani *et al.* have demonstrated that the use of antibiotics 8 weeks before to 4 weeks after the initiation of immunotherapy has been associated with lower ORR and poor clinical outcomes with significantly reduced PFS^[74]^. Data are lacking regarding the duration and timing of antibiotics that could impact the clinical outcomes of ICI. Increased stress-related β-adrenergic signaling has been shown to create an immunosuppressive TME and promote tumor growth^[77]^.

The role of cytoreduction nephrectomy in relation to ICI response remains relatively unestablished. Currently, there are no phase III clinical trials that have comprehensively evaluated the impact of cytoreductive nephrectomy on ICI response in RCC. A meta-analysis indicated that cytoreductive nephrectomy did not exhibit a significant association with the response to ICI treatments^[78]^.

The main mechanisms of resistance to ICIs in RCC are discussed in Table 1. All the above factors contribute to the response of tumors to ICI, and by understanding the molecular mechanisms behind the resistance to ICI, we can identify potential targets for therapeutic interventions to sensitize tumors to ICI in RCC. The main mechanisms of resistance to ICIs and major trials addressing the mechanisms of resistance in RCC are given in Table 1.

**Table 1:** Mechanisms of resistance in renal cell carcinoma

**Mechanisms of resistance**	**Main components involved in resistance**	**Major trials addressing resistance to ICI**
Tumor microenvironment	CD8+ T-cells	JAVELIN RENAL 101 exploratory analysis^[50]^, NCT03013335
	T-regulatory cells	
	Myeloid-derived suppressor cells	
	Tumor-associated macrophages	NCT03013335
	Memory T-cells	
	Cytokines	
Oncogenic pathways	IFN-γ signaling	NCT03010176
	MAPK pathway	
	Wnt/β-catenin pathway	
Immune checkpoints	PD-1	TITAN-RCC, TiNivo-2 (NCT04987203)
	PD-L1	CONTACT-03
	CTLA-4	NCT03849469, TITAN-RCC
	TIM3	NCT03652077, NCT02608268
	LAG3	NCT02996110, NCT02996110, NCT03849469, NCT00351949
	BTLA	
	TIGIT	NCT03119428
Other factors	Hypoxia	NCT03634540, NCT04195750, NCT03634540
	Gut microbiome	NCT03829111, GETUG-AFU 26 NIVOREN multicenter phase II study^[79]^

BTLA: B and T lymphocyte attenuator; CTLA-4: cytotoxic T lymphocyte-associated protein 4; ICI: immune checkpoint inhibitor; IFN-γ: interferon-γ; LAG3: lymphocyte-activation gene 3; MAPK: mitogen-activated protein kinase; PD-1: programmed death-1; PD-L1: programmed death ligand-1; RCC: renal cell carcinoma; TIGIT: T cell immunoglobulin and ITIM domain; TIM3: T cell immunoglobulin and mucin domain 3.

### Strategies to overcome resistance to immune checkpoint inhibitors

As mentioned above, the mechanisms of resistance to ICI are diverse, including the intra (PD-1 expression, TMB, *etc.*) and extra-tumoral factors (T-cell types and their infiltration, T-cell activation status, TAM, *etc.*). The main strategies that are utilized to overcome the primary resistance and prevent the acquired resistance to ICI are depicted below.

### Combination of treatments

One of the main strategies that is used to overcome resistance to ICI is a combinatorial approach. The combination of anti-PD-1 nivolumab and anti-CTLA-4 ipilimumab ICIs has already been approved and used with excellent clinical benefit in mRCC^[10]^. The clinical benefit was observed to be higher in the combination of ICIs compared with ICI monotherapy. The ORR with nivolumab monotherapy was 25% (refractory RCC population) and that of the ipilimumab/nivolumab combination was 42% (treatment naïve RCC population). Similarly, better OS was observed with the combination therapy when used in the first-line setting; OS of ipilimumab/nivolumab therapy in intermediate and poor-risk mRCC patients was 48.1 months (55 months in the ITT) and that of nivolumab monotherapy was 25 months (second line)^[9-11]^. CTLA-4 inhibition has been involved in the priming of T cells, depleting the immunosuppressive Tregs in the TME and anti-PD-1 has a role in reversing T cell exhaustion^[80]^.

Another strategy that is used to tackle acquired resistance to ICI is rechallenging the tumors after losing the response to ICI. One of our mRCC patients had a response for 18 months and single-agent pembrolizumab was discontinued for cutaneous IRAE. This response was maintained off therapy for another 18 months and then had progressive disease. We rechallenged the patient with single-agent pembrolizumab and observed a partial response again prior to a treatment holiday. This was consistent with loss of response rather than resistance formation. Although we have observed this in clinical practice, the accurate mechanism behind response on rechallenge is not completely understood. In metastatic melanoma, rechallenging patients with primary and secondary resistance to ICI have shown to have better therapeutic outcomes. In addition, the escalation of treatment (ICI combined with additional agents) has been shown to have higher response rates in melanoma^[81]^. A phase II study of salvage ipilimumab/nivolumab by Atkins *et al.* in mRCC has shown only modest benefit and is associated with increased side effects^[82]^. The benefit of Salvage ipilimumab and nivolumab in mRCC patients with primary and acquired resistance was analyzed in the TITAN-RCC trial^[83]^. They observed that half of the patients who received salvage ipilimumab/nivolumab for progression of the disease had clinical benefits (PR/CR 18% and SD 30%). This was irrespective of the timing of resistance development. In the recently published preliminary results of the phase III CONTACT-03 multicenter trial, patients with locally advanced or mRCC who progressed on prior ICI were randomized to receive atezolizumab and cabozantinib *vs.* cabozantinib alone. The addition of atezolizumab to cabozantinib did not lead to improved clinical outcomes. The median PFS in the atezolizumab and cabozantinib arm was 10.6 months (95%CI 9.8-12.3) compared to 10.8 months (95%CI 10.0-12.5) in the cabozantinib arm (HR = 1.03, *P* = 0.78). Similarly, the median OS in the combination arm was 25.7 months (95%CI 21.5-not evaluable) and was not evaluable in the cabozantinib arm (HR = 0.94, *P* = 0.69). The toxicities were higher in the combination arm, with 17 deaths compared to 9 deaths in the cabozantinib arm^[84]^. Another ongoing randomized phase III trial, TiNivo-2 trial, compares the effect of the combination of nivolumab and tivozanib with tivozanib monotherapy in patients who progressed on at least one line of ICI (NCT04987203)^[85]^. More biomarker studies are required to effectively understand the role of rechallenge/escalation strategy in mRCC without increasing the immune-related toxicities^[86]^. It would be ideal if such studies incorporated a measure of the immune status, which could ideally include factors of tumor (real-time), T-cell/ TAM, T-cell receptor repertoire, and other relevant host factors.

ICIs are combined with several targeted agents to enhance their efficacy. Several combinations of TKIs are approved in combination with ICIs, as mentioned previously. The addition of cabozantinib to nivolumab in the first-line setting in mRCC, as noted in the Checkmate 9ER, resulted in prolonged overall survival than that was observed with sunitinib alone. The OS with the combination was 49.5 months and that of sunitinib was 35.5 months. The ORR was significantly higher with the combination (55.7% with cabozantinib + nivolumab: 28% with sunitinib), which points to the fact that the combination of TKIs and nivolumab has an additive effect^[9,14,87]^. VEGF inhibitors have been studied in combination with ICIs and are shown to reverse resistance to ICI to a certain extent by regulating the immunosuppressive TME^[88]^. VEGF induces various changes in the TME, including the upregulation of PD-L1, PD-1, and CTLA-4 expression on dendritic cells and other immune cells, leading to immune exhaustion^[89]^. VEGF also elevates the proportion of MDSCs and T-regs in the TME, fostering an immunosuppressive environment^[90,91]^. Moreover, it impedes the differentiation of CD8+ T cells and exerts an inhibitory impact on effector T-cells^[92]^. When TKIs, which inhibit VEGF, are combined with ICIs, they reverse these immunosuppressive effects of VEGF, thereby enhancing T-cell priming and promoting the cytotoxic activities of immune cells^[93]^.

The combination of VEGF inhibitor bevacizumab and anti-PD-L1 atezolizumab has been studied in mRCC, and it was found that the combination increases CD8 T-cell density in the tumors and aids in antigen-specific T-cell migration^[94]^. Unfortunately, this clinical trial was negative and failed to demonstrate meaningful improvement in survival.

### Targeting TME components

As immunosuppressive TME is a major factor causing resistance to ICIs, several components of the TME such as MDSCs, Tregs, and cytokines can be targeted to potentially reverse the resistance. As mentioned above, several cytokines play a role in recruiting immunosuppressive cells and CTLs into the TME. Epigenetic silencing of CXCL9 and CXCL10 expression can lead to resistance to ICI by reduction of the tumor-infiltrating lymphocytes^[43]^. In an ovarian cancer model, epigenetic modulator therapy reversed the suppression of these cytokines and improved response to ICIs^[95]^. In metastatic urothelial cancer, the inhibition of TGF-β is found to be associated with improved response to ICIs^[96]^.

### Targeting immune exhaustion markers

The overexpression of the immune exhaustion marker such as TIM-3, and LAG-3 leads to resistance to ICI therapy as mentioned above. TIM-3 has been reported as a predictive biomarker of ICI response in RCC. In RCC, co-expression of TIM3 and PD-1 correlated with large tumor size, aggressive phenotype, lower PFS, and OS and showed a higher risk of relapse^[97]^. Therefore, targeting these exhaustion markers alone or in combination with PD-1 can lead to better clinical response. There are several ongoing clinical trials studying the effect of anti-TIM3 antibodies alone and in combination with anti-PD-1/PD-L1 antibodies in advanced solid tumors and hematological malignancies (NCT02817633, NCT03099109). The benefit of anti-LAG3 has been extensively investigated in solid tumors including mRCC. A phase I trial evaluating the utility of a recombinant soluble LAG-3 fusion protein, IMP321, showed an acceptable toxicity profile and better clinical outcomes^[98]^. There are several ongoing clinical trials evaluating the efficacy of relatlimab, an anti-LAG3 antibody in combination with ICI in advanced solid tumors, including mRCC (NCT02996110, NCT05328908).

Cancer vaccines incorporating dendric cells, tumor-specific peptides, and oncolytic virus therapy have been investigated in various tumors. These have been found to induce antigen presentation and T-cell priming^[99]^. A clinical trial investigating a multipeptide vaccine with nivolumab has demonstrated immunologic activity with promising survival in high-risk melanoma^[100]^. Clinical trials investigating oncolytic virus therapy in advanced solid tumor malignancies are ongoing (NCT03206073, NCT05346484). A phase II trial investigating pexa-vec, an oncolytic vaccinia virus, has shown a 76% disease control rate including one CR in patients with mRCC^[101]^. Given the benefit of fecal microbiota transplant in germ-free mice to overcome resistance to PD-1 blockade, gut microbiota modulation is emerging as a strategy to improve the efficacy of ICI and reverse resistance^[73]^. As stress pathways and β-adrenergic signaling play a significant role in the enhancement of an immunosuppressive TME, thereby decreasing the clinical response of ICI, blocking of the adrenergic pathways would enhance the activity of ICI. This was evidenced in a phase I clinical trial in which the addition of propranolol, a β-blocker to pembrolizumab in metastatic melanoma, showed promising clinical activity^[102]^.

## CONCLUSION AND FUTURE DIRECTIONS

With the use of ICI, the survival of patients with mRCC has improved significantly. Although many patients have achieved durable clinical benefits, including CR, primary and acquired resistance to ICI is a significant challenge that remains to be addressed. This resistance may be due to various factors, such as alterations in the tumor microenvironment, activation of alternative signaling pathways, or overexpression of immune exhaustion markers. Thus, there is an unmet need to elucidate the mechanisms underlying immunotherapy resistance in metastatic renal cell carcinoma and to develop strategies to overcome this issue. Addressing this problem is crucial to improve the efficacy of immunotherapy and ultimately improve outcomes for patients with metastatic renal cell carcinoma.

## DECLARATIONS

### Copyright

© The Author(s) 2023.
